# Molecular Characterization of a Reemergent *Brugia malayi* Parasite in Sri Lanka, Suggestive of a Novel Strain

**DOI:** 10.1155/2021/9926101

**Published:** 2021-08-07

**Authors:** C. H. Mallawarachchi, T. G. A. N. Chandrasena, G. P. Withanage, R. Premarathna, S. M. N. S. M. Mallawarachchi, Nilmini Y. Gunawardane, Ranil S. Dasanayake, Dinesh Gunarathna, N. R. de Silva

**Affiliations:** ^1^Medical Research Institute, Colombo, Sri Lanka; ^2^Faculty of Medicine, University of Kelaniya, Ragama, Sri Lanka; ^3^Ministry of Health and Indigenous Medicine, Colombo, Sri Lanka; ^4^University of Colombo, Colombo, Sri Lanka; ^5^Ministry of Plantation Industries, Battaramulla, Colombo, Sri Lanka

## Abstract

Sri Lanka achieved elimination status for lymphatic filariasis in 2016; still, the disease remains a potential public health issue. The present study is aimed at identifying a subperiodic *Brugia sp.* parasite which has reemerged in Sri Lanka after four decades via molecular-based analysis. Polymerase chain reaction performed with pan-filarial primers specific for the internal transcribed spacer region-2 (ITS-2) of the rDNA of *Brugia* filarial parasites isolated from human, canine, and feline blood samples yielded a 615 bp band establishing the species identity as *Brugia malayi*. Comparison of the ITS2 sequences of the reemerged *B. malayi* isolates with GenBank sequences revealed a higher sequence homology with *B. pahangi* than *B. malayi* with similar phylogenetic evidence. However, the mean interspecies Kimura-2-parameter pairwise divergence between the generated *Brugia* sequences with *B. malayi* and *B. pahangi* was less than 3%. During the analysis of parsimony sites of the new ITS2 sequences, substitutions at A36T, A296G, T373A, and G482A made the sequences different from both *B. pahangi* and *B. malayi* suggesting the possibility of a new genetic variant or a hybrid strain of *B. malayi* and *B. pahangi.* Mosquito dissections and xenomonitoring identified *M. uniformis* and *M. annulifera* as vectors of this novel strain of *B. malayi* circulating among cats, dogs, and humans in Sri Lanka.

## 1. Introduction

Lymphatic filariasis (LF) is a neglected tropical disease estimated to have affected 40 million people worldwide, with an at-risk population of 893 million people residing in 49 endemic countries [1]. Although infection is not fatal, it is a leading cause of permanent disability and was targeted for elimination as a public health problem by 2020 [1].

Three species of filarial worms, namely, *Wuchereria bancrofti*, *Brugia malayi* (nocturnally periodic strain), and *Brugia timori*, are known to cause classical lymphatic filariasis in humans. Of the three human filarial parasites, *W. bancrofti* is the most prevalent, causing 90% of infections worldwide, while the rest are attributed to *B. malayi* (prevalent in Southeast Asia and in South-Western parts of India) and to a lesser extent *B. timori* (limited to the islands of Timor-Leste in eastern Indonesia) [1]. A zoonotic strain of *B. malayi* (subperiodic strain), which is a natural parasite of a variety of wild and domestic animals (cats, dogs, monkeys, and slow lorises), has been documented to cause accidental zoonotic infections in humans [2–5]. Serological and molecular evidence of human infections by another zoonotic filarial worm, *B. pahangi* (a natural parasite of felines), has been reported from Kuala Lumpur, with clinical manifestations consistent with LF [6]. These zoonotic filariae (subperiodic *B. malayi* and *B. pahangi*) also contribute to the human disease burden especially in United States, South America, and Southeast Asian region [2, 3, 7].

Both *W. bancrofti* and *B. malayi* (nocturnally periodic strain) were prevalent in Sri Lanka in the past [8]. Vector control activities focused on clearance of aquatic vegetation (a requirement for breeding of *Mansonia* sp. mosquitoes) resulted in clearance of *B. malayi* infections from the country in late nineteen sixties [8]. Subsequently, five rounds of single annual mass drug administration (MDA) were implemented from 2002 to 2006 in the three provinces (Western, North-Western, and Southern) endemic for Bancroftian filariasis using diethylcarbamazine citrate (300 mg) combined with albendazole (400 mg). In 2016, Sri Lanka was categorized as a country that had eliminated LF as a public health problem, following fulfilment of critical criteria stipulated by the World Health Organization (WHO) for the verification of elimination [9]. However, surveillance activities during the phases of post-MDA and postelimination revealed the sporadic occurrence of brugian filariasis in all three LF endemic provinces [10–12]. Periodicity studies revealed that the reemergent *Brugia* species microfilaria (MF) exhibited nocturnal subperiodicity, implying a strain of different origin [12]. Zoonotic surveillance in areas affected by human brugian filariasis has revealed a high prevalence of *B. malayi* microfilaraemia among dogs and cats [13]. The existence of another zoonotic filarial species, *Brugia ceylonensis*, among local canines has also been documented in the past [14].

Exact speciation of the reemerged *Brugia* parasite was important as zoonotic *B. malayi*, *B*. *pahangi*, and *B. ceylonensis* have all been implicated in accidental human infections [3, 4, 6, 15]. The morphological similarity of MF of *Brugia* sp. necessitated a molecular-based approach (PCR and sequencing) for species identification [15].

Studies on the genetic variability of *B. malayi* indicate that parasite isolates from different regions diverged significantly [16]. Gene sequences of zoonotic *Brugia* species are sparse in major data bases, and with increasing human infections, there will be an increased need for genomic repositories [17]. Such information may support the genus or species clarifications based on consensus gene targets. Thus, sequencing of the *Brugia* sp. MF that were isolated from humans and animals of Sri Lanka was carried out to construct the phylogeny and assess their relationship to other *Brugia* species.

Knowledge of the transmission dynamics of the newly emerged parasite is important for control. The vector preferences of the reemerged parasite may differ from the nocturnal periodic strain of *B. malayi* documented in the past; thus, entomological investigations were performed for clarification of the vector species.

Against this background of events, a comprehensive analysis of the reemerged *Brugia* sp. parasite of Sri Lanka, as to its species identity, the phylogeny and genetic relatedness to other *Brugia* species and its vector preferences were studied using parasitological and molecular methods.

## 2. Methods

### 2.1. Human Sample Collection and DNA Extraction

**Figure 1:**
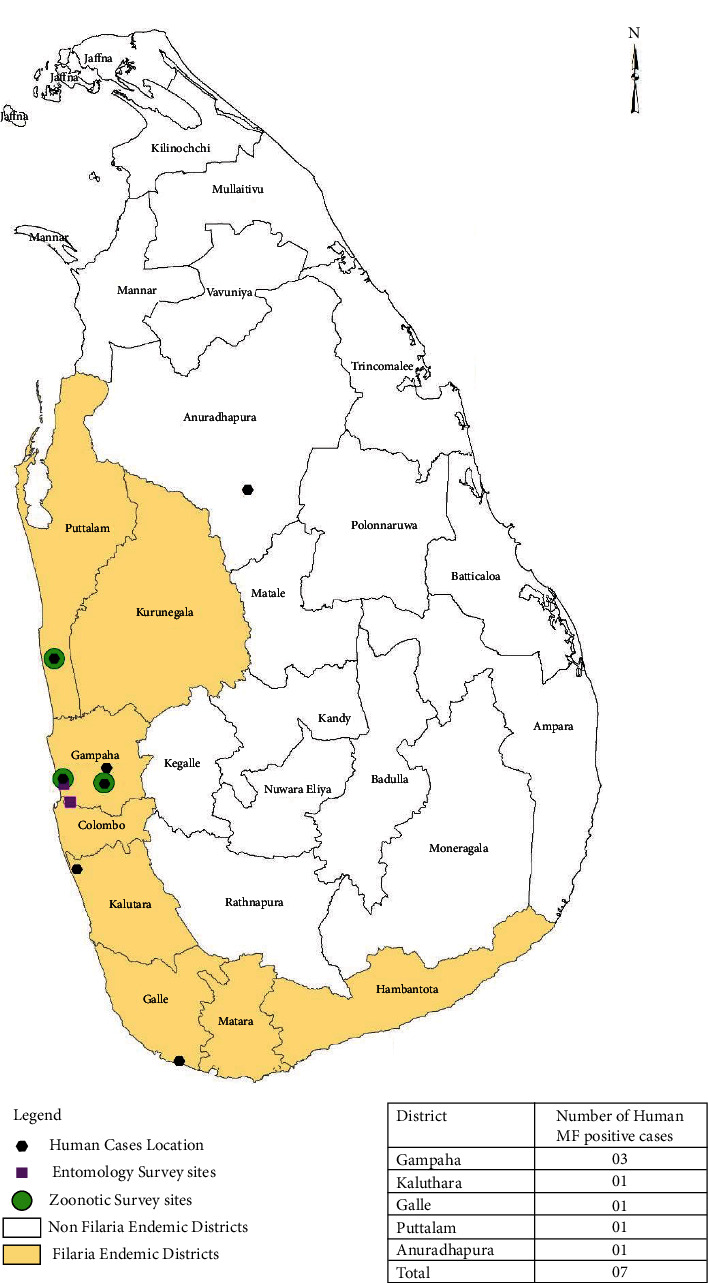
Human brugian cases and survey locations (Zoonotic and Entomology).

A total of seven human blood samples positive for *Brugia* sp. MF by thick night blood smears (NBS) during the study period were included to the study. Positive samples were collected from four districts (Gampaha, Kalutara, Puttalam, and Galle) of the three endemic provinces, and one was from a case detected from a nonendemic district in the North Central province (Anuradhapura) (Figure 1). Nine blood samples positive for *Brugia* antibodies by the Brugia rapid test that were detected during an initial survey were also included in the molecular analysis [12]. The samples were stored at -20°C in EDTA prior to DNA extraction. The MF in blood was concentrated by Nuclepore® membrane filtration, and the filter membranes with trapped MF were used for DNA extraction. The DNA was extracted using the ReliaPrep™ Blood DNA Miniprep System (Catalogue number A5082) according to the manufacturer's instructions [18] with some modifications.

Briefly, a volume of 180 *μ*l of phosphate-buffered saline was added to polycarbonate membranes with MF and incubated for 45 minutes while shaking (300 rpm). A volume of 20 *μ*l proteinase K and 200 *μ*l cell lysis buffer solution was added to the content and incubated at 56°C for 10 minutes to which 250 *μ*l of binding buffer was added. The content was then transferred to ReliaPrep™ binding columns and centrifuged at 8,000 rpm. The binding columns were transferred to fresh collection tubes, and 500 *μ*l of column wash solution was added and centrifuged for three minutes at 8,000 rpm and the follow through was discarded. After repeating the former step twice, the binding columns were finally eluted with 50 *μ*l nuclease-free water. The DNA concentrations were measured by fluorometry according to the instructions (Manual #TM396: http://www.promega.com/protocols/) and stored at -20°C until used.

### 2.2. DNA Amplification

The procedure stated by Rishniw et al. was used with some modifications using Promega reagents [19]. The pan-filarial primers (DIDR-F1 5′-AGT GCG AAT TGC AGA CGC ATT GAG-3′ and DIDR-R1 5′-AGC GGG TAA TCA CGA CTG AGT TGA-3′) that spanned the ITS2 of the rDNA designed by Rishniw et al. were employed to amplify the target DNA region. Known positive and negative controls were included in each PCR reaction. The PCR procedure consisted of an initial heating step at 94°C for 2 minutes, 32 cycles of denaturation at 94°C for 30 seconds, annealing at 58°C for 30 seconds, and extension at 72°C for 30 seconds and a final extension at 72°C for 7 minutes and a soak at 4°C in an Applied Biosystems 2720 Thermal Cycler (Thermo Fisher Scientific).

Discrimination of the six species was based on the size of the amplified PCR products. DIDR-F1 and DIDR-R1 primers amplified 484 bp, 542 bp, 578 bp, 584 bp, 615 bp, and 664 bp products from *Dirofilaria repens*, *Dirofilaria immitis*, *Acanthocheilonema reconditum*, *Acanthocheilonema dracunculoides*, *B. malayi*, and *B. pahangi*, respectively [19].

### 2.3. Canine and Feline Sample Processing

The zoonotic surveillance for filarial parasites was done at three locations in the districts of Gampaha (Weliweriya and Wattala) and Puttalam (Madampe) of Western and Northwestern provinces, respectively (Figure 1). Molecular speciation of the zoonotic *Brugia* parasite by PCR using panfilarial primers specific for ITS2 region confirmed its identity as *B. malayi* as detailed in a previous publication (13).

### 2.4. Mosquito Vector Analysis

#### 2.4.1. Sample Collection, DNA Extraction, and Amplification

The mosquito surveillance was carried out in the district of Gampaha in the same areas where zoonotic surveillance was done, using one cattle-baited net trap in each location (Figure 1). Mosquitoes of *Mansonia* species were identified using morphological keys. Heads and thoraces of all *Mansonia* species mosquitoes were dissected to identify filarial larvae.

DNA of filarial larvae was extracted using the MightyPrep reagent (Takara Bio Inc., Japan) according to the manufacturer's guide with some modifications as follows: the mosquito heads and thoraces that were positive for filarial larvae were crushed and mounted temporarily on glass slides and flushed with 200 *μ*l of MightyPrep reagent into a microcentrifuge tube. The lysate was homogenized by a hard vortex for 10 seconds. New pipette tips were used each time to prevent cross contamination of samples. The lysates were incubated at 95°C for 10 minutes. Subsequently, the sample lysates were cool down to room temperature. The cooled lysates were hard-vortexed for another 10 seconds. Finally, the samples were centrifuged at 12,000 rpm for 10 minutes and stored at −20°C until used for PCR. DNA amplification was done using the same set of primers and procedure used for human and animal samples.

#### 2.4.2. DNA Sequencing

The Sanger method [20] was applied in cycle sequencing of amplicons (ITS2 region of rDNA) of MF in blood samples of humans (*n* = 2), canines (*n* = 3), and felines (*n* = 6). Sample selection was based on the concentration of the DNA extracts.

#### 2.4.3. Homology Comparison

The forward and reverse chromatograms were assembled in Lasergene 8.00 software suite (DNASTAR Inc., USA). BLASTn tool was used to compare the nucleotide similarity of study sequences with those available in the NCBI GenBank database. Consensus sequences were aligned using MAFFT 7, and phylogenetic tree was developed using the Neighbor-Joining (NJ) algorithm executed in MEGA-X software [21], and the tree was constructed with K2P substitution model and gamma distributed rates. Bootstrap analysis of 1,000 replicates was performed to determine the robustness of clades. To develop the tree, 22 reference DNA sequences were retrieved from the GenBank database. Meantime, pairwise distances were calculated between individuals in each species using the MEGA-X software. Further, polymorphisms in the alignment were analyzed using the DnaSP (version 6.12.01 x64) software.

### 2.5. Ethical Clearance

Ethical approval for the study was obtained from the Ethics Review Committees of the Faculty of Medicine University of Kelaniya (P/108/09/2016) and the Medical Research Institute (40/2016).

## 3. Results

**Figure 2:**
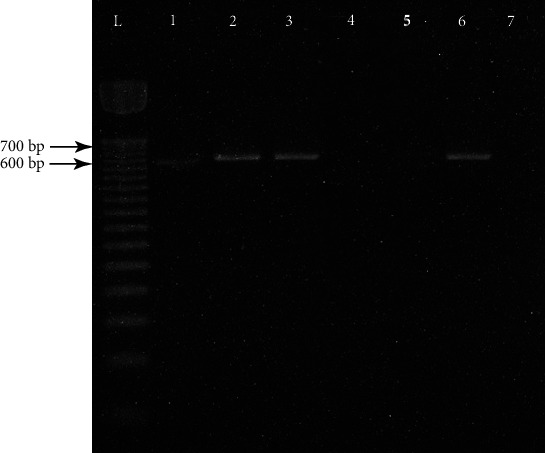
Gel electrophoresis of the PCR products of human samples. The gel image shows the results of the PCR amplification of samples 1 to 7 using Pan-filarial primers (Lanes 1 to 5: human samples, Lane 6: positive control, Lane 7: negative control, and Lane L: 50 bp DNA marker).

**Figure 3:**
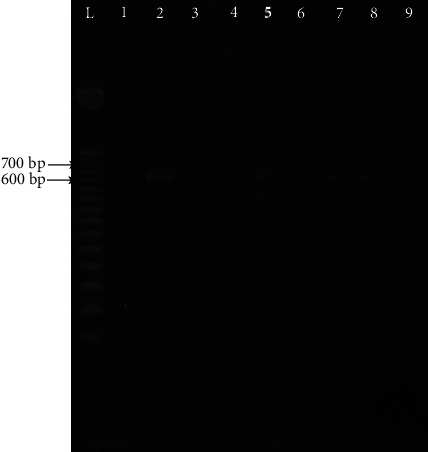
Gel electrophoresis of pan-filarial primer-specific PCR amplicons of human, canine, and feline samples from the zoonotic study area Pubudugama. The gel image shows the results of the pan-filarial primer-specific PCR amplification of samples 1 to 9 (Lanes 1: human sample, Lane 2 to 4: dog samples, Lane 5 to 7: cat samples, Lane 8: positive control, Lane 9: negative control, and Lane L: 50 bp DNA marker).

Six of the seven human blood samples were positive for *Brugia* sp. MF by NBS that were tested by PCR produced a band of 615 bp specific for *B. malayi* (Figure 2). The sample that failed to elicit a PCR band had very low microfilaraemia (1MF/slide). All human blood samples that were only positive for anti-Brugia antibodies by BRT (*n* = 9) failed to elicit a PCR band. The zoonotic samples also elicited bands of 615 bp specific for *B. malayi* (Figure 3).

A total of 82 *Mansonia* sp. mosquitoes were identified in the cattle-baited net trap collection, namely, *M. annulifera* (65), *M. uniformis* (14), and *M. indiana* (3). Filarial larval stages were detected in 20.73% (17/82) of the dissected *Mansonia* mosquitoes. Of them, 7.14% (1/14) and 24.6% (16/65) were *M. uniformis* and *M. annulifera*, respectively. The DNA extracts of all infected *Mansonia* mosquitoes elicited the 615 bp band of *B. malayi* when analysed by pan-filarial primer-specific PCR.

## 4. Phylogenetic Analysis

**Figure 4:**
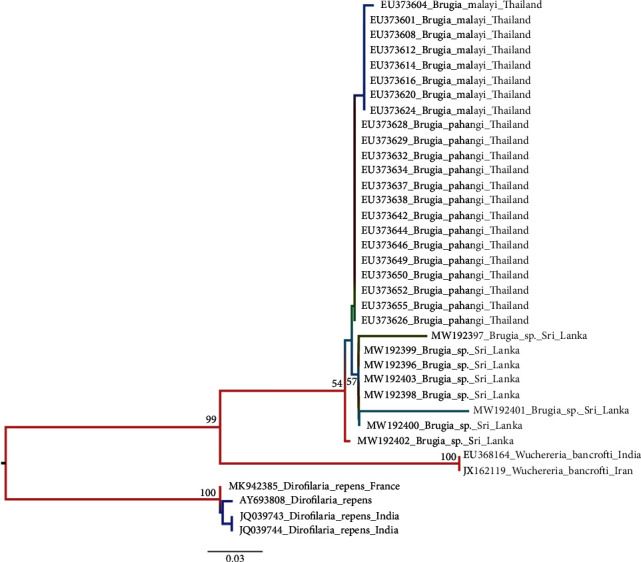
Phylogenetic tree constructed from partial rDNA sequencing (ITS2 region); 14 sequences of *B. pahangi* and 8 sequences of *B. malayi* were retrieved from GenBank and are shown with accession numbers. Phylogenetic tree was constructed using MEGA-X software with 1,000 bootstrap support (MW192396-8—Cat, Madampe; MW192399—Human, Pubudugama; MW192400—Human, Weliweriya; MW192401—Dog, Pubudugama; MW192402-3—Cat, Pubudugama).

Homology analysis performed on GenBank using the BLASTN of NCBI and the amplified ITS2 sequences of the *B. malayi* MF isolated from humans, dogs, and cats in this study showed the sequences to have higher homology with *B. pahangi* than *B. malayi* (Figure 4). Homology analysis of developed sequences from different host species was compared, and the highest estimated evolutionary divergence of *B. malayi* MF was observed between dogs and cats (2.7%) and the lowest between humans and cats (0.7%). The estimated evolutionary divergence of *B. malayi* MF specimens between humans and dogs was 1.9% (Annex 1).

Phylogenetic analyses of the generated ITS2 gene sequences from human, canine, and feline blood samples were clustered with *B. pahangi* and *B. malayi.* All the *Brugia* sp. ITS2 sequences demonstrated a monophyletic origin from the same ancestor and formed separate clusters (Figure 4). The lengths of the horizontal lines are proportional to the minimum number of nucleotide differences required to join nodes. The mean K2P divergence analysis of the new ITS2 sequences showed less than 3% phylogenetic divergence with other studied brugian sequences (Table 1). The interspecies K2P divergence between new sequences and *B. malayi* was 2.41% while that of for *B. pahangi* is 1.8%.

During the polymorphism analysis, 21 parsimony informative sites with one deletion were identified and nine (09) of them were crucial polymorphic sites in the ITS2 gene alignment (Table 2). The results indicated that the new sequences were similar to both *B. pahangi* and *B. malayi*. This is similar to the Neighbor-Joining phylogenetic tree inferred using the Tajima-Nei method with 500 replicates bootstrap support. The new sequences exhibited similarities to *B. malayi i*n 277 and 288 positions while showing differences at A99T and A118G with 176delA showing similarities to *B. pahangi.* However, the new sequences also demonstrated fixed substitutions at A36T, A296G, T373A, and G482A which made the new sequences different from *B. pahangi.* Therefore, the new sequences may probably be a variant or hybrid strain of both *B. malayi* and *B. pahangi*.

## 5. Discussion

PCR confirmed that the reemerged subperiodic *Brugia* parasite was the same as that circulating among dogs and cats in the region. The phylogenetic analysis of the ITS2 region of the rDNA of both human and zoonotic parasites indicated that the reemerged *B.malayi* parasites had a close resemblance to *B. pahangi.* However, the K2P distance between the new sequences and available previous sequences was less than 3%, the cut-off for nematode species demarcation [22, 23].

The presence of a novel zoonotic *Brugia* strain closely related to *B. malayi* and *B. pahangi* has been previously queried on molecular characterization of the rDNA sequences of a filarial nematode and comparison with known gene sequences in the GenBank. These filarial nematodes were isolated from the inguinal lymph nodes of a patient based in the city of New York with an extensive travel history to Central America and the Caribbean [24].

A canine survey in Kerala had reported the presence of *B. malayi* like MF that had elicited a histochemical staining pattern consistent with that of *B. malayi*. The Hha1 primer PCR product of this *B. malayi* like parasites was cloned and sequenced (2 clones, Accession numbers JN 601136 and JN 601137), and the phylogeny revealed that the *B. malayi* like parasite was genetically closer to *B. pahangi* suggesting the existence of a genetic variant closely related to *B. malayi* and *B. pahangi* in the natural environment [25].

Further, the new sequences demonstrated 2.4% and 1.8% genetic divergence with *B. malayi* and *B. pahangi*, respectively. This interspecies genetic distance is much higher than the distance difference between *B. malayi* and *B. pahangi*, which is 0.5%. Similar low levels of interspecies divergence have been reported previously in ITS-2-based studies of hookworms from northern fur seals and California sea lions [23].

The possibility of this variant strain of *B. malayi* being the species documented as *B. ceylonensis* requires due consideration. The adult filarial nematodes of *B. ceylonensis* were first described in 1962 in the lymphatic glands of dogs from Sri Lanka. It was documented as a novel species closely related to *B. patei* based on morphological characteristics [26]. However, genomic data of *B. ceylonensis* is not available in GenBank database for comparison with the reemerged *B. malayi.*

The results of the present study indicate that the variant strain is well established among animals with occurrence of spill-over infections among humans. This variant *B. malayi* appears to have a limited capacity to infect humans as case numbers were relatively low compared to the heavy zoonotic reservoirs of infection in Sri Lanka [13]. This could be attributed to low transmissibility owing to vector characteristics (low affinity of *Mansonia* sp. mosquitoes for human blood) or the enhanced immune response generated by a poorly adapted zoonotic parasite conferring natural resistance to infection. The rising number of cases in the recent past may be indicative of the parasite's potential to evolve and adapt to humans or may reflect the research interest generated by the PELF.

Sentinel surveillance of animals and xenomonitoring may serve in defining populations at risk of infection. Thus, vector identification is important not only for implementing the appropriate vector control measures but also for mapping the distribution of infection. *M. annulifera* and *M. uniformis* are zooanthropophagic mosquito species in Sri Lanka that were implicated in the transmission of periodic *B. malayi* in the past [27, 28]. This study confirmed their capability of transmitting the variant *B. malayi* (subperiodic) in Sri Lanka.

ITS regions 1 and 2 have been used by many investigators for studies on phylogenetic reconstruction, genetic variability, and divergence of closely related taxa of a wide range of organisms [29–31]. The molecular characterization of the MF of this variant *B. malayi* isolated from humans was limited to two samples, and analysis was focused only on the ITS2 region of the rDNA. A more comprehensive genome-wide analysis of rDNA as well as mitochondrial DNA of MF and adult stages of this variant *B. malayi* may be required for clarification of the taxonomy of the parasite. Further studies on *B. malayi* parasites isolated from humans and animals from different geographical locations in the country as well as comprehensive entomological surveys which cover a wide array of mosquito species are indicated to characterize the novel variant zoonotic *B. malayi.*

## 6. Conclusion

The reemerged *B. malayi* parasite in Sri Lanka appears to be a novel genetic variant. Domestic canines and felines were identified as the zoonotic reservoirs and Mansonia sp. mosquitoes (*M. annulifera* and *M. uniformis*) were implicated as vectors. This reemerged parasite could well pose a threat to the LF elimination status of the country because vectors and infectious zoonotic reservoirs are present in abundance. The application of molecular identification techniques will be invaluable for clarification of taxonomy, epidemiology, and ecology of this novel variant strain of *B. malayi.*

## Figures and Tables

**Table 1:** Interspecific genetic distances among the analysed species.

	SL_G1	*B. malayi*	*B. pahangi*	*W. bancrofti*	*D. repens*
SL_G1	0.0				
*B. malayi*	2.414	0.0			
*B. pahangi*	1.801	0.589	0.0		
*W. bancrofti*	22.53	21.074	20.309	0.0	
*D. repens*	33.359	31.996	31.113	37.115	0.0

**Table 2:** Crucial polymorphic sites observed in the study sequences.

Position	Study sequences	*B. malayi*	*B. pahangi*
36	T	A	A
99	T	A	T
118	G	A	G
176	—	A	—
277	T	T	—
282	G	G	A
296	G	A	A
373	T	A	A
482	G	A	A

## Data Availability

The datasets supporting the conclusions of this article are included in the manuscript.

## Supplementary Materials

Supplementary Materials Aneex 1 Homology analysis of developed sequences from different host species.
