# Supplementary material for: Molecular Characterization of a Reemergent Brugia malayi Parasite in Sri Lanka, Suggestive of a Novel Strain
Source: Biomed Res Int. 2021 Aug 7;2021:9926101. doi: 10.1155/2021/9926101 (PMC8370822; doi:10.1155/2021/9926101)
Supplement: Supplementary Materials — Aneex 1 Homology analysis of developed sequences from different host species. [file 9926101.f1.docx]

**Annex 1 - Homology analysis of developed sequences from different host species**


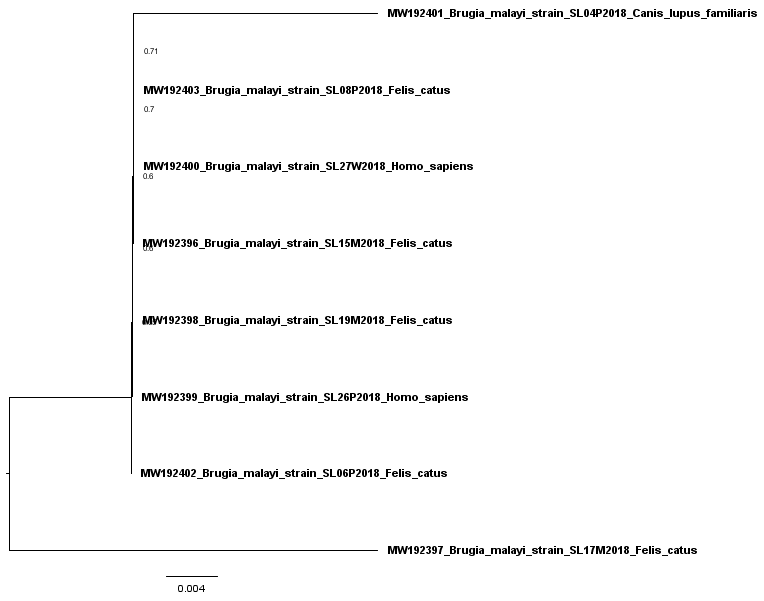


Figure A1. Homology analysis of partial rDNA sequencing, (ITS2 region); Phylogenetic tree was constructed using MEGA-X software with 1,000 bootstrap support. Analyses were conducted using the Kimura 2-parameter model. This analysis involved 8 nucleotide sequences at total of 270 positions in the final dataset. The sequences were clustered together except for the MW192397. *Homo sapiens* (humans), *Felis catus* (cats), and *Canis lupus familiaris* (dogs)

In the analysis of estimated evolutionary divergence of *B. malayi* MF specimens from different host species, the highest was observed between dogs and cats (2.7%) and the lowest between humans and cats (0.7%). The estimated evolutionary divergence of *B. malayi* MF specimens between humans and dogs was 1.9%.
